# Beyond BMI: independent and opposing effects of overweight and obesity and triglycerides on 90-day functional outcomes after acute ischemic stroke

**DOI:** 10.3389/fneur.2026.1782157

**Published:** 2026-04-13

**Authors:** Tao Xie, Qianqian Xiao, Zhenni Peng, Hengna Li, Chenrui Song, Yuanyuan Wang, Shi Wang, Fei Zhou, Qi Fang

**Affiliations:** 1 Department of Neurology, The First Affiliated Hospital of Soochow University, Suzhou, China; 2 Department of Neurology, The Third Affiliated Hospital of Naval Medical University, Shanghai, China

**Keywords:** body mass index, ischemic stroke, modified Rankin Scale, obesity, overweight, prognosis, triglycerides

## Abstract

**Objective:**

To investigate whether the “obesity paradox” in acute ischemic stroke (AIS) is a masking effect of metabolic lipid reserves. We evaluated the independent and opposing associations of body weight status and admission triglycerides (TG) with 90-day functional outcomes to distinguish the structural burden of obesity from metabolic health.

**Methods:**

This dual-center retrospective cohort study included 571 consecutive AIS patients recruited between 2019 and 2024. Patients were categorized into normal-weight (NW, *n* = 245; BMI 18.5–23.9 kg/m^2^) and overweight-or-obesity (OW, *n* = 326; BMI ≥ 24.0 kg/m^2^) groups. The primary endpoint was an excellent functional outcome [modified Rankin Scale (mRS) 0–1] at 90 days. Multivariable logistic regression and inverse probability weighting (IPW) were employed to isolate the independent effects of weight status and TG levels.

**Results:**

At 90 days, the proportion of patients achieving an excellent outcome was significantly higher in the NW group than in the OW group (60.4% vs. 50.6%; *p =* 0.020). In univariable analysis, patients who achieved an excellent outcome (mRS 0–1) had significantly higher admission TG levels than those who did not [median 1.33 (IQR 0.97–1.92) vs. 1.13 (IQR 0.90–1.48) mmol/L; *p <* 0.001]. After adjusting for comprehensive confounders including age, NIHSS, and other lipid profiles, overweight-or-obesity was independently associated with lower odds of an excellent outcome (adjusted OR = 0.611, 95% CI: 0.394–0.945; *p =* 0.027). Conversely, higher admission TG levels were significantly associated with better recovery (adjusted OR = 1.405 per 1 mmol/L increase, 95% CI: 1.057–1.867; *p =* 0.019). These opposing associations remained robust in IPW sensitivity analyses.

**Conclusion:**

The “obesity paradox” in AIS appears to be a masking effect driven by TG reserves. Once disentangled from metabolic benefits, overweight and obesity emerge as independent risk factors for poorer recovery. These findings support a phase-specific metabolic management strategy: mitigating the physical and systemic burdens of obesity while ensuring sufficient TG levels within the physiological range to support neural repair during the acute window.

## 1 Introduction

The global rise in obesity constitutes a major public health challenge, with well-established links to cardiovascular and cerebrovascular diseases (1). While obesity is a clear risk factor for stroke incidence, its impact on post-stroke recovery remains controversial (2). Some studies report an “obesity paradox,” where overweight or mildly obese patients exhibit better survival or functional outcomes (3). However, others contradict these findings, suggesting that excess weight impedes recovery (4). These inconsistencies likely stem from the limitations of using body mass index (BMI) as a sole metric, as it fails to capture the underlying metabolic heterogeneity or nutritional status of individual patients.

Clinically, distinguishing between “structural burden” and “metabolic health” is crucial for individualized management. BMI does not differentiate between adiposity and muscle mass, nor does it reflect metabolic reserves that may be vital during the catabolic stress of acute stroke. Serum triglycerides (TG), traditionally viewed strictly as a vascular risk factor, may also reflect the availability of essential energy and structural lipid reserves during the acute phase of illness. We hypothesize that the observed “obesity paradox” may, in fact, be a masking effect, where the protective role of TG-mediated metabolic reserves conceals the inherent detrimental impact of excess body weight.

To address this knowledge gap, we conducted a dual-center retrospective cohort study involving 571 Chinese AIS patients. Our primary objective was to isolate the independent effects of body weight status and serum TG on 90-day functional outcomes. By applying rigorous statistical adjustments and inverse probability weighting (IPW), we sought to determine whether overweight and obesity—once isolated from their associated metabolic benefits—independently hinder recovery. We hypothesized that while higher BMI imposes a physical and pro-inflammatory burden, higher TG levels signal a metabolic reserve beneficial for neural repair, thereby exerting opposing influences on functional recovery.

## 2 Methods

### 2.1 Study population

#### 2.1.1 Setting and timeframe

This study was conducted at the stroke centers of the First Affiliated Hospital of Soochow University and the Third Affiliated Hospital of Naval Medical University. The study period spanned January 2019 to December 2024.

#### 2.1.2 Participant selection

We performed a retrospective analysis of prospectively collected data over the study period. A total of 571 eligible patients were included from 597 screened cases according to predefined inclusion and exclusion criteria.

#### 2.1.3 Inclusion criteria

- Age ≥18 years;
- (2) Acute ischemic stroke (AIS) patients categorized by body mass index (BMI) into two groups: normal weight (NW), 18.5 ≤ BMI < 23.9 kg/m^2^; and overweight-or-obese (OW/OB; BMI ≥ 24.0 kg/m^2^;
- (3) Neuroimaging confirmation of an acute ischemic lesion within 7 days of symptom onset by magnetic resonance imaging (MRI) or computed tomography (CT).

#### 2.1.4 Exclusion criteria

(1) Intracerebral hemorrhage or intracranial mass lesion (including hemorrhagic transformation detected after admission requiring urgent non-contrast head CT to exclude);
(2) Severe infection or septic shock;
(3) Malignancy with an expected survival ≤90 days;
(4) Pregnancy.
(5) Underweight status (BMI < 18.5 kg/m^2^), which did not meet the predefined BMI eligibility criteria for the present analysis.

All patients (or their legally authorized representatives) provided informed consent, and data were analyzed in de-identified form. The study protocol was approved by the Ethics Committee of the First Affiliated Hospital of Soochow University (approval No. 2021052) and by the Ethics Committee of the Third Affiliated Hospital of Naval Medical University (approval No. EHBHKY2025-K101-P001).

### 2.2 Data collection

#### 2.2.1 Baseline clinical characteristics

From medical records we abstracted age, sex, smoking history, and prior medical conditions (hypertension, diabetes mellitus, prior stroke, and atrial fibrillation [including pre-existing AF or new-onset AF during the index hospitalization]). Initial bedside measurements included height, weight, body mass index (BMI), systolic blood pressure (SBP), diastolic blood pressure (DBP), first (admission) blood glucose, and the National Institutes of Health Stroke Scale (NIHSS) score.

#### 2.2.2 Definitions of comorbidities

Hypertension was defined as a documented diagnosis or use of antihypertensive medication, or SBP ≥ 140 mmHg and/or DBP ≥ 90 mmHg at admission. Diabetes mellitus was defined as a documented diagnosis or use of glucose-lowering therapy, or admission fasting plasma glucose ≥7.0 mmol/L and/or HbA1c ≥ 6.5%. BMI was calculated as weight (kg) divided by height squared (m^2^).

#### 2.2.3 Laboratory measurements

Laboratory variables included first-available triglycerides (TG) drawn within 24 h of admission (primarily in a non-fasting state), total cholesterol, high-density lipoprotein cholesterol (HDL-C), low-density lipoprotein cholesterol (LDL-C), serum albumin, serum creatinine, and fibrinogen.

#### 2.2.4 Perfusion imaging processing and analysis

Multimodal CT, including CT perfusion (CTP) when available, was used solely to support acute reperfusion decision-making according to local protocols; CTP-derived parameters were not incorporated into the statistical analyses of this study.

### 2.3 Therapeutic interventions

For patients with suspected stroke within 24 h of onset (or last known well), neurologists performed multimodal CT—non-contrast CT (NCCT), CT angiography, and CT perfusion. Treatment decisions—including intravenous thrombolysis (standard-dose alteplase 0.9 mg/kg or low-dose 0.6 mg/kg), endovascular thrombectomy, bridging therapy, or standardized medical management without reperfusion—were made by senior, experienced stroke neurologists.

### 2.4 Outcomes

Functional status at 90 days was assessed by trained personnel using the modified Rankin Scale (mRS) via in-person visit or structured telephone interview. Good outcome was defined as mRS 0–2 and poor outcome as mRS 3–6; excellent outcome was defined as mRS 0–1 and non-excellent outcome as mRS 2–6 (5).

### 2.5 Statistical analysis

Normality of continuous variables was examined with the Kolmogorov–Smirnov test. Non-normally distributed continuous variables are presented as median (interquartile range, IQR) and compared with the Mann–Whitney U test; normally distributed variables are expressed as mean ± standard deviation (SD) and compared with independent-samples t tests. Categorical variables are summarized as counts (percentages) and compared using the χ^2^ test or Fisher’s exact test, as appropriate. Analyses were performed with SPSS (IBM SPSS Statistics for Windows, version 26.0) and GraphPad Prism (version 9.0.0, GraphPad Software, San Diego, CA, United States). Two-sided *p* values <0.05 were considered statistically significant.

A multivariable logistic regression model was constructed to identify independent predictors of excellent functional outcome (mRS 0–1) at 90 days. Age, sex, baseline NIHSS score, acute treatment modality (intravenous thrombolysis, mechanical thrombectomy, conservative management), BMI group (normal weight vs. overweight-or-obesity), and serum TG level (per 1 mmol/L increase) were entered as covariates. In a sensitivity analysis, we additionally fitted an extended multivariable model further adjusted for hypertension, diabetes mellitus, atrial fibrillation, smoking status, and admission random glucose.

To evaluate potential bias from loss to follow-up or missingness, we conducted an inverse probability weighting (IPW) sensitivity analysis. A logistic regression model predicting inclusion in the primary analysis (yes = 1, no = 0) was fitted with age, sex, and baseline NIHSS as predictors to derive stabilized IPW (sIPW) for each patient. Covariates were selected *a priori* based on coverage and relevance to minimize row-wise deletion due to excessive missingness. The main multivariable model was then re-estimated using these weights to assess the robustness of the primary findings.

## 3 Results

### 3.1 Study flow

**Figure 1:**
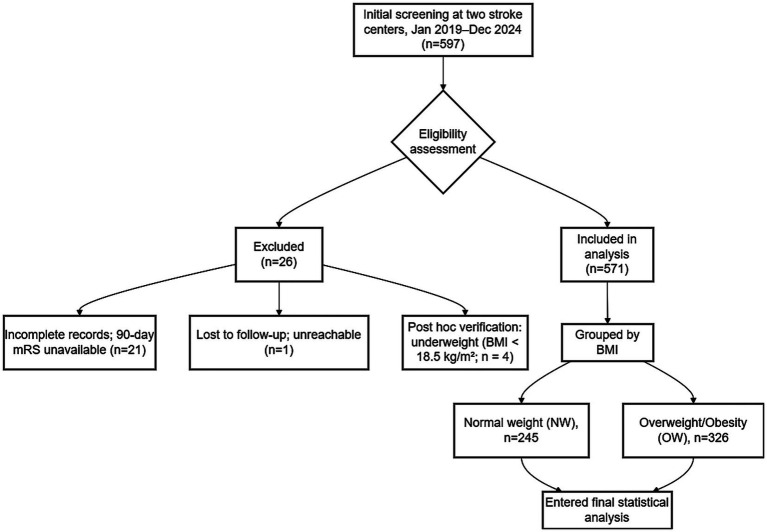
Study flow diagram. A total of 597 consecutive patients with acute ischemic stroke were screened across two clinical centers from January 2019 to December 2024. Following eligibility assessment, 571 patients met the inclusion criteria and were categorized by body mass index (BMI) into either the normal-weight group (NW, *n* = 245) or the overweight-or-obesity group (OW, *n* = 326). Twenty-six patients were excluded based on the following criteria: incomplete clinical records or missing 90-day modified Rankin Scale (mRS) scores (*n* = 21), loss to follow-up (*n* = 1), and ineligibility identified during *post hoc* verification (*n* = 4).

A total of 597 consecutive patients with acute ischemic stroke were screened for eligibility. Of these, 571 (95.6%) completed the 90-day mRS assessment and were included in the final analysis. According to the Chinese BMI thresholds, patients were categorized into the normal-weight group (NW, 18.5–23.9 kg/m^2^; *n* = 245) and the overweight-or-obesity group (OW, ≥24.0 kg/m^2^; *n* = 326). Reasons for exclusion (*n* = 26, 4.4%) included incomplete medical records (*n* = 21), loss to follow-up (*n* = 1), and underweight status (BMI < 18.5 kg/m^2^; *n* = 4) (Figure 1).

### 3.2 Baseline characteristics and outcomes

Compared with the NW group, patients in the OW group were significantly younger [median 65.0 (IQR 54, 72) vs. 69.0 (IQR 61, 75) years; *p* < 0.001] and had a higher proportion of males (72.1% vs. 64.1%, *p* = 0.041). While weight and BMI were markedly higher in the OW group (*p <* 0.001), height also showed a slight difference (median 168 vs. 165 cm; *p =* 0.005).

Regarding vascular risk factors, the OW group exhibited a significantly higher prevalence of hypertension (73.0% vs. 61.6%; *p* = 0.004) and current/ever smoking (28.8% vs. 20.0%; *p* = 0.016). In contrast, atrial fibrillation (25.3% vs. 16.9%, *p* = 0.013) and cardioembolic etiology (TOAST classification; *p* = 0.140 for overall subtype distribution) were more common in the NW group. Baseline stroke severity was comparable between the two groups [median NIHSS 6 (IQR 3, 11) in the OW group vs. 5 (IQR 3, 10) in the NW group; *p* = 0.093]. Specifically, the distribution of stroke severity—categorized as mild (NIHSS 0–4), moderate (5–15), and severe (≥ 16)—was similar between the groups (*p* = 0.407), with NW and OW patients showing mild (46.5% vs. 41.1%), moderate (40.8% vs. 44.5%), and severe stroke (12.7% vs. 14.4%), respectively.

In terms of metabolic profiles, the OW group demonstrated significantly higher admission random glucose (*p* = 0.004) and serum triglyceride levels [median 1.35 (IQR 1.00, 1.86) vs. 1.07 (IQR 0.86, 1.49) mmol/L; *p* = 0.001]. Other lipid parameters, including total cholesterol, HDL-C, and LDL-C, did not differ significantly between groups. Acute treatment strategies, such as intravenous thrombolysis and endovascular thrombectomy, were well-balanced between the NW and OW groups (*p* = 0.789) (Table 1).

**Table 1:** Baseline characteristics and outcomes in normal-weight vs. overweight-or-obese patients.

Characteristic	Normal weight (NW), *n* = 245	Overweight-or-Obesity (OW), *n* = 326	*p*-value
Demographics			
Age, years, median (IQR)	69 (61, 75)	65.0 (54, 72)	<0.001*
Male, *n* (%)	157 (64.1)	235 (72.1)	0.041*
Height, cm, median (IQR)	165 (160, 170)	168 (160, 172)	0.005*
Weight, kg, median (IQR)	60.0 (55.0, 65.0)	75.0 (69.0, 80.0)	<0.001*
BMI, kg/m^2^, mean ± SD	22.0 ± 1.4	26.9 ± 2.3	<0.001*
BMI categories, *n* (%)			
Normal weight (18.5–23.9)	245 (100.0)	0 (0.0)	
Overweight (24.0–27.9)	0 (0.0)	245 (75.2)	
Obesity (≥28)	0 (0.0)	81 (24.8)	
*Subgroup: BMI ≥ 30.0*	0 (0.0)	31 (9.5)	
Clinical parameters			
SBP, mmHg, median (IQR)	150 (133, 168)	152 (137, 172)	0.023*
DBP, mmHg, median (IQR)	83 (74, 95)	87 (78, 100)	<0.001*
Baseline NIHSS, median (IQR)	5 (3, 10)	6 (3, 11)	0.093
*Subgroup: Stroke Severity, n (%)*			0.407
Mild (0–4)	114 (46.5)	134 (41.1)	
Moderate (5–15)	100 (40.8)	145 (44.5)	
Severe (≥16)	31 (12.7)	47 (14.4)	
Laboratory values			
Random glucose, mmol/L, median (IQR)	6.5 (5.5, 8.3)	7.0 (5.9, 9.3)	0.004*
Fibrinogen (Fbg), g/L, median (IQR)	3.1 (2.6, 3.7)	3.1 (2.6, 3.7)	0.594
Creatinine, μmol/L, median (IQR)	67.0 (58.0, 84.0)	69.4 (58.9, 82.7)	0.555
Triglycerides, mmol/L, median (IQR)	1.07 (0.86, 1.49)	1.35 (1.00, 1.86)	0.001*
Total cholesterol, mmol/L, median (IQR)	4.24 (3.64, 4.98)	4.22 (3.59, 4.94)	0.542
HDL-C, mmol/L, median (IQR)	1.07 (0.89, 1.28)	0.97 (0.85, 1.15)	0.418
LDL-C, mmol/L, median (IQR)	2.67 (2.02, 3.24)	2.68 (2.09, 3.32)	0.490
Albumin, g/L, mean ± SD	40.22 ± 5.91	40.77 ± 5.28	0.235
Medical and personal history, *n* (%)			
Hypertension	151 (61.6)	238 (73.0)	0.004*
Diabetes mellitus	62 (25.3)	96 (29.4)	0.274
Current/ever smoking	49 (20.0)	94 (28.8)	0.016*
Atrial fibrillation	62 (25.3)	55 (16.9)	0.013*
TOAST subtype, *n* (%)			0.140
LAA	115 (46.9)	168 (51.5)	
CE	53 (21.6)	47 (14.4)	
SAO	60 (24.5)	95 (29.1)	
OC	11 (4.5)	11 (3.4)	
SUE	6 (2.4)	5 (1.5)	
Treatment strategy, *n* (%)			0.789
Intravenous thrombolysis	90 (36.7)	113 (34.7)	
Endovascular thrombectomy	29 (11.8)	44 (13.5)	
Standard medical therapy	126 (51.4)	169 (51.8)	
Effectiveness			
90-day mRS, median (IQR)	1 (0, 3)	1 (0, 3)	0.088
90-day excellent outcome (mRS 0–1), *n* (%)	148 (60.4)	165 (50.6)	0.020*
90-day good outcome (mRS 0–2), *n* (%)	177 (72.2)	218 (66.9)	0.169

Data are presented as median (interquartile range, IQR), mean ± standard deviation (SD), or *n* (%), as appropriate. NW, normal weight; OW, overweight/obesity; BMI, body mass index; SBP, systolic blood pressure; DBP, diastolic blood pressure; NIHSS, National Institutes of Health Stroke Scale; HDL-C, high-density lipoprotein cholesterol; LDL-C, low-density lipoprotein cholesterol; Fbg, fibrinogen; TOAST, Trial of ORG 10172 in Acute Stroke Treatment; LAA, large-artery atherosclerosis; CE, cardioembolism; SAO, small-artery occlusion; OC, other determined etiology; SUE, stroke of undetermined etiology; mRS, modified Rankin Scale.

### 3.3 Functional outcomes and multivariable analyses

**Figure 2:**
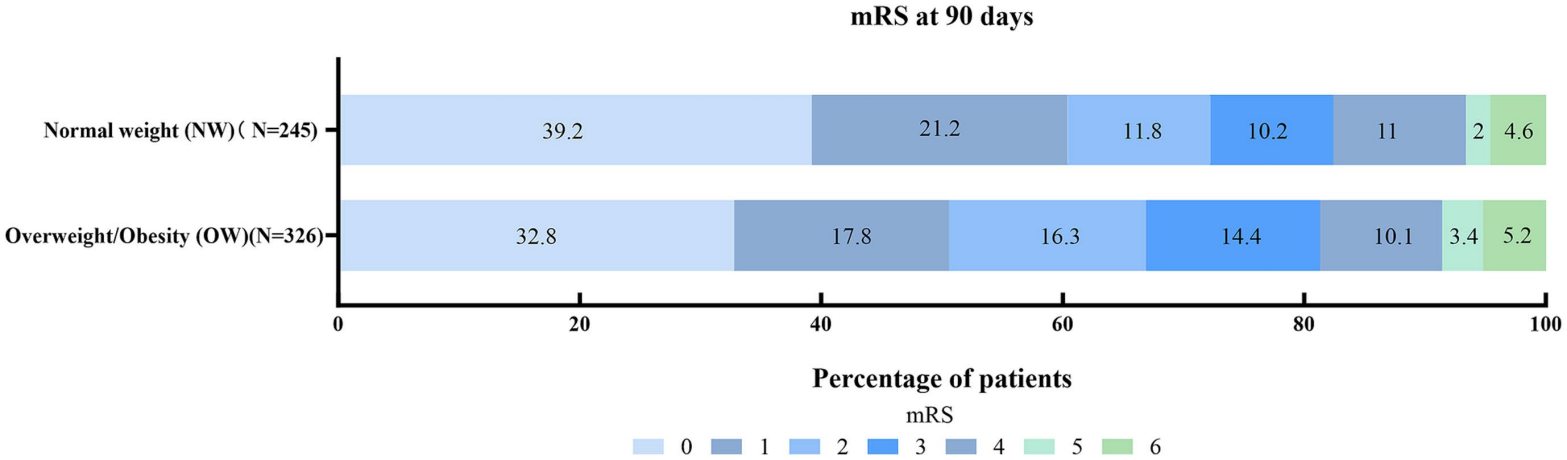
Distribution of 90-day modified Rankin Scale (mRS) scores by weight status. The stacked horizontal bar chart illustrates the proportions of patients in each mRS category (0–6) at 90 days, comparing the normal-weight group (NW, *n* = 245) with the overweight-or-obesity group (OW, *n* = 326). Each color-coded segment represents a distinct mRS score, with the gradient from lighter to darker blue indicating better to worse functional outcomes (mRS 0–6, respectively). Numeric labels within each segment denote the percentage of patients in that specific category. While the overall ordinal distribution across the full spectrum of mRS scores did not reach statistical significance between the two groups (*p* = 0.088 by Mann–Whitney U test), the proportion of patients achieving an excellent functional outcome (mRS 0–1) was significantly higher in the NW group compared to the OW group (60.4% vs. 50.6%, *p* = 0.020). Vertical dashed gridlines at 20% increments are displayed to facilitate visual comparison.

At 90 days, the overall distribution of mRS scores was statistically similar between the NW and OW groups (*p =* 0.088) (Table 1, Figure 2). Overall, 313 (54.8%) patients in the entire cohort achieved an excellent functional outcome (mRS 0–1) at 90 days. Regarding the primary endpoint, the proportion of patients achieving an excellent outcome (mRS 0–1) was significantly higher in the NW group compared to the OW group (60.4% vs. 50.6%; *p =* 0.020). In univariable analysis, patients who achieved an excellent outcome (mRS 0–1) had significantly higher admission TG levels compared to those with a poor outcome [median 1.33 (IQR 0.97–1.92) vs. 1.13 (IQR 0.90–1.48) mmol/L; *p <* 0.001]. The rate of good outcome (mRS 0–2) was also higher in the NW group, although the difference did not reach statistical significance (72.2% vs. 66.9%; *p =* 0.169) (Table 1).

**Figure 3:**
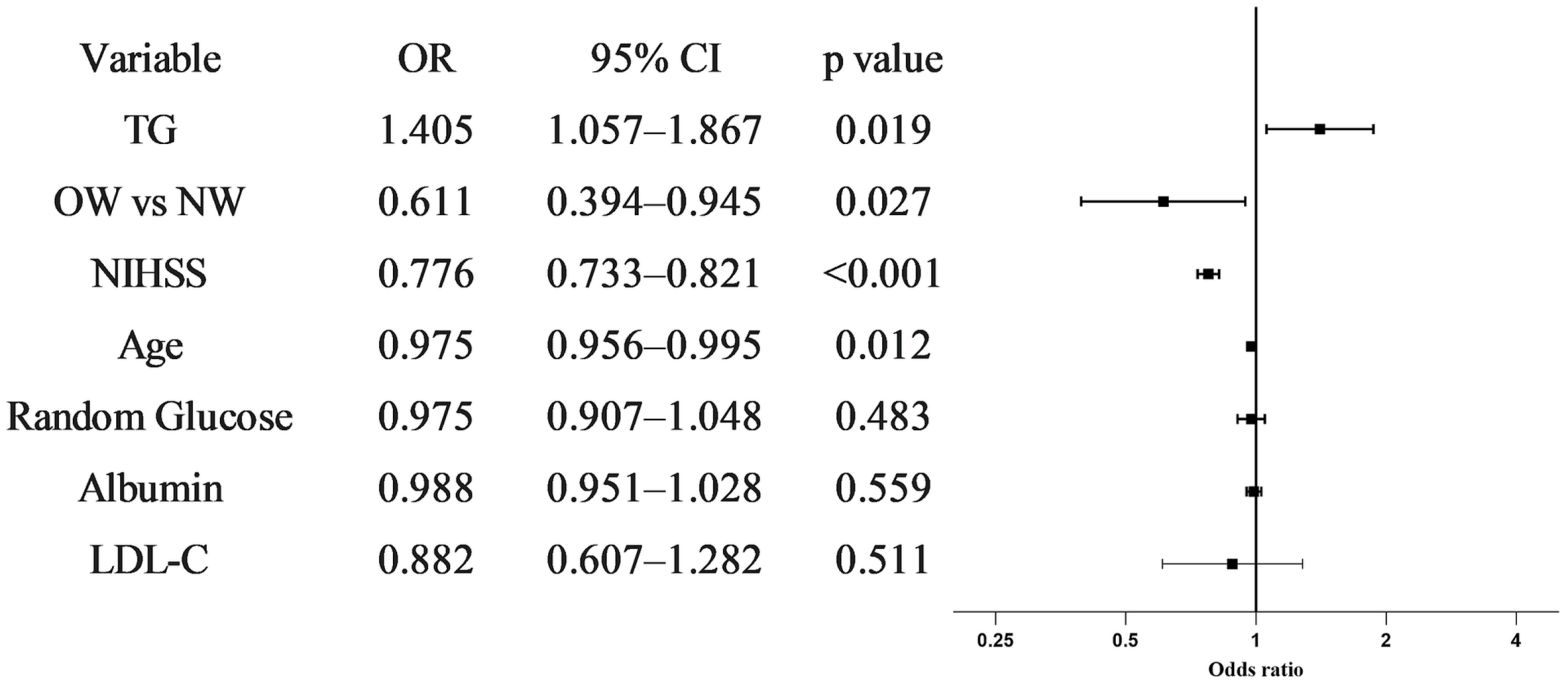
Multivariable logistic regression for predictors of excellent 90-day outcome (mRS 0–1). The forest plot illustrates the independent and opposing associations of body weight status and admission triglyceride (TG) levels with an excellent functional outcome (mRS 0–1). Odds ratios (ORs, represented by squares) and 95% confidence intervals (CIs, represented by horizontal lines) are derived from the extended multivariable model. This model was rigorously adjusted for age, sex, baseline NIHSS score, medical history (hypertension, diabetes mellitus, smoking, and atrial fibrillation), treatment strategies (IVT, MT, or medical treatment), TOAST subtypes, random glucose, serum albumin, and a comprehensive lipid profile (total cholesterol, HDL-C, and LDL-C). The vertical dashed line at OR = 1.0 represents the null value. Overweight or obesity (OW) was independently associated with a lower likelihood of an excellent outcome, whereas higher admission TG levels (per 1 mmol/L increase) served as a significant independent predictor of better recovery.

To evaluate the independent associations of body weight and TG with outcomes, we constructed an extended multivariable logistic regression model (Supplementary Table S1). After adjusting for age, sex, baseline NIHSS score, medical history (hypertension, diabetes mellitus, smoking, atrial fibrillation), treatment strategies, TOAST subtypes, random glucose, albumin, and a comprehensive lipid profile (total cholesterol, HDL-C, and LDL-C), overweight-or-obesity remained independently associated with lower odds of an excellent outcome (adjusted OR = 0.611, 95% CI: 0.394–0.945; *p =* 0.027). Conversely, higher admission TG levels were independently associated with better recovery (adjusted OR = 1.405 per 1 mmol/L increase, 95% CI: 1.057–1.867; *p =* 0.019) (Figure 3). Older age and higher baseline NIHSS were also significant independent predictors of poor outcome.

The robustness of these findings was confirmed through two sensitivity analyses. First, using a good outcome (mRS 0–2) as an alternative endpoint, the associations remained consistent in direction, with TG maintaining a positive trend for recovery (OR = 1.388, 95% CI: 0.980–1.967; *p =* 0.065) (Supplementary Table S2). Second, after applying inverse probability weighting (IPW) to account for potential selection bias, the results remained stable: OW status was significantly associated with lower odds of excellent outcome (IPW-adjusted OR = 0.611; *p =* 0.021), while higher TG levels remained a significant protective factor (IPW-adjusted OR = 1.384; *p =* 0.010) (Supplementary Table S3).

## 4 Discussion

In this dual-center cohort, we observed that body weight status and admission triglyceride (TG) levels exert independent and opposing effects on 90-day functional outcomes in acute ischemic stroke (AIS) patients. Overweight or obesity (OW) was independently associated with a lower likelihood of achieving an excellent functional recovery (mRS 0–1), whereas higher TG levels demonstrated a significant protective effect. The overall excellent outcome rate in our cohort (54.8%) and the rate in the normal-weight subgroup (60.4%) fall within the range of outcomes reported in recent Chinese stroke cohorts, which vary from 49.4% in more severe subgroups to 86.7% in optimally selected minor stroke patients (6, 7).

The “obesity paradox”—the observation that overweight patients sometimes fare better after stroke—has been consistently reported, but these studies predominantly focused on mortality rather than functional recovery. Vemmos et al. found that overweight and obese patients had significantly lower 10-year mortality (8), and a systematic review confirmed this survival advantage across multiple studies (9). In the Chinese population, Zhao et al. similarly reported that overweight was associated with favorable functional recovery at three months (10). However, a closer examination of these studies reveals that the relationship between BMI and post-stroke outcomes is more complex than a simple “protective” effect. Liu et al. demonstrated that while higher BMI linearly improves survival, its relationship with favorable functional outcomes follows a U-shaped pattern—suggesting that the determinants of mortality and disability after stroke are fundamentally different (11).

Our study extends this line of inquiry by further dissecting functional recovery into two distinct levels: functional independence (mRS 0–2) and excellent recovery (mRS 0–1). We found that the protective effect of TG was specific to the higher threshold of excellent recovery, a distinction that previous studies—which did not account for metabolic reserves—could not capture. This suggests that what appeared as an “obesity paradox” in earlier studies may reflect a confounding of two separate phenomena: the survival benefit of higher BMI (possibly driven by nutritional reserve) and the functional benefit of metabolic reserves (reflected by TG). Once these are disentangled, the detrimental impact of excess weight on high-quality neurological recovery becomes apparent.

The mechanism underlying this phenomenon can be understood as a statistical confounding effect. Overweight patients typically present with relatively higher TG levels, and when this metabolic trait is not fully adjusted for in multivariable models, it may offset or even conceal the vascular damage and pro-inflammatory risks inherent to adipose tissue (2, 12). When these effects are separated through regression models that include both weight and lipid factors, the detrimental impact of obesity on excellent functional recovery is unmasked. These results suggest that in the prognostic evaluation framework of stroke, it is essential to consider the patient’s “weight load” and “metabolic reserve” as two relatively independent dimensions.

The positive association between TG and stroke prognosis may initially appear to stand in tension with conventional clinical wisdom, as hypertriglyceridemia is a traditional risk factor for atherosclerosis. However, during the acute phase following a stroke, its biological role may undergo a stage-specific transition. Clinical observations show that lower admission TG levels are often associated with more severe neurological deficits (i.e., higher NIHSS scores) (13), indicating a correlation between lipid reserves and initial stroke severity.

From a pathophysiological perspective, TG may serve as a critical metabolic reserve during the acute stress phase. First, as a highly concentrated energy substrate, free fatty acids (FFAs) released from TG hydrolysis can provide an emergency energy supply to the ischemic penumbra, helping to delay the occurrence of early neurological deterioration (END) (14). Second, recent prospective studies have shown that higher TG levels are associated with a reduced risk of stroke-associated pneumonia (SAP) (15, 16). The possible mechanism is that lipid metabolism participates in maintaining immune cell function, and sufficient lipid reserves help protect the integrity of the respiratory mucosal barrier, thereby reducing the risk of infectious complications. For stroke patients, a relatively stable early clinical course—an uneventful recovery window—is a crucial prerequisite for subsequent high-quality neurological reconstruction.

Another noteworthy finding in this study is that the supportive effect of TG on prognosis is more pronounced at the level of “excellent recovery” (mRS 0–1) than at “functional independence” (mRS 0–2). This disparity may hold significant biological connotations.

We speculate that achieving functional independence primarily depends on the success of early revascularization and the control of core infarct volume—factors more related to acute management and the initial degree of injury. In contrast, reaching a state of complete asymptomatic recovery (mRS 0–1) requires a higher level of neurological repair, including axonal sprouting, synaptic remodeling, cortical reorganization, and myelin repair (14). These processes demand far more energy and structural lipids than what is required for basic neuronal survival. In our study, the median TG level of patients achieving an excellent outcome was 1.33 mmol/L (IQR 0.97–1.92), significantly higher than that of the non-excellent outcome group (1.13 mmol/L). This level represents a robust metabolic state within the normal physiological range (<1.7 mmol/L) defined by latest 2026 ACC/AHA/AACVPR/ABC/ACPM/ADA/AGS/APhA/ASPC/NLA/PCNA Guideline on the Management of Dyslipidemia (17). These findings suggest that sufficient triglyceride reserves within the physiological range may serve as a vital metabolic buffer during the post-stroke hypercatabolic phase, providing the essential substrates necessary for high-quality neuroplasticity.

Mechanistically, the protective role of TG is not limited to energy supply. PUFAs released from TG hydrolysis are substrates for mitochondrial *β*-oxidation and essential raw materials for the restructuring of neuronal membranes. More importantly, these fatty acids serve as precursors for the synthesis of Specialized Pro-resolving Mediators (SPMs) involved in the resolution of inflammation (18, 19). These mediators can actively regulate the inflammatory microenvironment around the infarct, inhibiting excessive inflammation and creating favorable conditions for neural repair. Furthermore, TG metabolism is regulated by the PPARs signaling pathway (20), the activation of which has been confirmed to protect the neurovascular unit after ischemia by improving blood–brain barrier function and inhibiting microglial overactivation. Collectively, the TG levels associated with excellent outcomes in our cohort—with a median of 1.33 mmol/L and an interquartile range of 0.97–1.92 mmol/L—were predominantly concentrated between approximately 1.3 and 1.7 mmol/L. Notably, this range lies entirely within the physiological threshold defined by the 2026 dyslipidemia guideline (<1.7 mmol/L), suggesting that the protective effect operates in the eulipidemic state rather than in the setting of hypertriglyceridemia. This observation points to an optimal metabolic window within the upper-normal range, where energy substrates for neuroplasticity are sufficient while lipotoxic risks are avoided.

It is imperative to clarify that the findings of this study should not be interpreted as a defense of hyperlipidemia or a negation of lipid-lowering therapy. In the long term, controlling lipid levels to delay the progression of atherosclerosis remains the cornerstone of primary and secondary stroke prevention. The 2026 AHA dyslipidemia guideline explicitly emphasizes intensive control strategies for LDL-C. For triglycerides, the goal of the guidelines is to maintain them within a normal physiological range rather than reducing them without limit (17).

Within this framework, our study offers a phase-specific clinical perspective. During the acute repair phase following a stroke—typically the first 2 to 4 weeks post-onset—maintaining sufficient TG levels within the physiological range may be important for meeting the high energy demands of neuronal repair (19). In clinical practice, patients are often in a state of low intake due to diagnostic requirements or the illness itself, and when combined with the enhanced catabolism caused by acute stress, TG levels may drop significantly in the short term. A mechanical application of the “lower is better” philosophy to all lipids during this window could inadvertently deprive the patient of the basic metabolic substrates required for neural repair.

Based on these insights, we propose a balanced, phase-specific management approach that distinguishes between the long-term goal of weight reduction and the acute need for energy preservation (21). On one hand, clinicians should address the physical and systemic loads imposed by obesity through early rehabilitation, airway management, and intervention for sleep-disordered breathing (22)—effectively “unloading” the patient’s metabolic burden. On the other hand, attention should be paid to optimizing nutritional support to avoid an excessive drop in TG levels due to unnecessary strict fasting, ensuring a continuous supply of essential fatty acids to fuel neurorehabilitation. In this context, admission TG levels—readily available from non-fasting samples—could serve as a simple biomarker to identify patients who may require more intensive nutritional or rehabilitation support (10, 17). This refined, phase-specific approach, conducted under the general principles of existing guidelines, may provide a new entry point for optimizing high-quality recovery in stroke patients.

### 4.1 Limitations

Our study has limitations inherent to its retrospective design. First, although IPW analysis suggests our results are robust to selection bias, residual confounding from unmeasured factors such as socioeconomic status, rehabilitation intensity, and pre-stroke diet cannot be excluded. Second, we relied on BMI, which cannot differentiate between visceral adiposity and muscle mass. The lack of data on waist circumference or body composition limits our ability to dissect the specific contribution of sarcopenic obesity versus pure adiposity. Third, TG was measured only at admission; dynamic changes in lipid profiles during hospitalization might offer additional prognostic value. Finally, the proposed immunometabolic mechanisms remain speculative and require validation through prospective studies incorporating inflammatory biomarkers and metabolomics.

## 5 Conclusion

In summary, overweight or obesity and serum triglycerides exert independent and opposing effects on 90-day functional outcomes in AIS patients. While excess body weight hinders the achievement of excellent recovery, higher circulating triglycerides appear to confer a protective metabolic benefit. Future research should integrate body composition analysis with metabolomics to refine these phenotypes, ultimately guiding precision nutrition and rehabilitation strategies.

## Data Availability

The data analyzed in this study is subject to the following licenses/restrictions: Access to the original datasets of this study is restricted due to the following reasons: Privacy and Confidentiality: The data are derived from hospital electronic medical records and contain sensitive, protected health information that could compromise patient privacy and confidentiality. Ethical and Legal Compliance: The study was conducted under specific ethical approvals (No. 2021052 and No. EHBHKY2025-K101-P001) which, together with Chinese regulations on personal information protection and medical data management, prohibit the public deposition of raw identifiable patient data. In accordance with these restrictions, the raw data are not publicly available. Access to the fully anonymized analytical dataset for the purpose of verifying the research findings may be granted upon reasonable and justified request to the corresponding author, subject to review and approval by the involved ethics committees and institutions, and the execution of a formal data use agreement. Requests to access the anonymized datasets underlying this study should be directed to the corresponding author: QF, Department of Neurology, The First Affiliated Hospital of Soochow University, Suzhou, China. Email: fangqi_008@126.com.

## Glossary

BMI: Body Mass Index
CE: Cardioembolism (TOAST subtype)
CI: Confidence Interval
CT: Computed Tomography
CTA: CT Angiography
CTP: CT Perfusion
DBP: Diastolic Blood Pressure
DXA: Dual-Energy X-ray Absorptiometry
Fbg: Fibrinogen
HDL-C: High-Density Lipoprotein Cholesterol
IQR: Interquartile Range
IPW: Inverse Probability Weighting
IVT: Intravenous Thrombolysis
LDL-C: Low-Density Lipoprotein Cholesterol
LAA: Large-Artery Atherosclerosis (TOAST subtype)
MRI: Magnetic Resonance Imaging
mRS: Modified Rankin Scale
MT: Mechanical Thrombectomy
NCCT: Non-Contrast CT
NIHSS: National Institutes of Health Stroke Scale
NW: Normal Weight
OC: Other Causes (TOAST subtype)
OR: Odds Ratio
OSA: Obstructive Sleep Apnea
OW: Overweight or obesity
PPAR: Peroxisome Proliferator-Activated Receptor
SAO: Small-Artery Occlusion (TOAST subtype)
SBP: Systolic Blood Pressure
SD: Standard Deviation
SUE: Stroke of Undetermined Etiology (TOAST subtype)
TC: Total Cholesterol
TG: Triglycerides
TOAST: Trial of ORG 10172 in Acute Stroke Treatment (etiologic classification)
