# Supplementary material for: Beyond BMI: independent and opposing effects of overweight and obesity and triglycerides on 90-day functional outcomes after acute ischemic stroke
Source: Front Neurol. 2026 Apr 13;17:1782157. doi: 10.3389/fneur.2026.1782157 (PMC13111007; doi:10.3389/fneur.2026.1782157)
Supplement: Supplementary file 1 [file Table_1.docx]

**Supplementary Table S1. Extended multivariable logistic regression model for excellent outcome (mRS 0–1) at 90 days.**

| **Variable** | **B** | **SE** | **OR** | **95% CI** | **p value** |
| --- | --- | --- | --- | --- | --- |
| **Exposure Variables** |  |  |  |  |  |
| TG | 0.340 | 0.145 | 1.405 | 1.057–1.867 | 0.019* |
| OW vs NW | -0.493 | 0.223 | 0.611 | 0.394–0.945 | 0.027* |
| **Other Lipid Profiles** |  |  |  |  |  |
| Cho | 0.146 | 0.187 | 1.157 | 0.802–1.669 | 0.435 |
| HDL-C | -0.014 | 0.054 | 0.986 | 0.887–1.097 | 0.798 |
| LDL-C | -0.126 | 0.191 | 0.882 | 0.607–1.282 | 0.511 |
| **Demographics and Severity** |  |  |  |  |  |
| Age | -0.025 | 0.01 | 0.975 | 0.956–0.995 | 0.012* |
| Male | 0.361 | 0.247 | 1.434 | 0.883–2.330 | 0.145 |
| NIHSS | -0.254 | 0.029 | 0.776 | 0.733–0.821 | <0.001* |
| **Metabolic and Nutritional Markers** |  |  |  |  |  |
| Random glucose | -0.026 | 0.037 | 0.975 | 0.907–1.047 | 0.483 |
| Albumin | -0.012 | 0.02 | 0.988 | 0.951–1.028 | 0.559 |
| **Treatment Strategy** |  |  |  |  | 0.275 |
| IVT vs. Med | 0.333 | 0.238 | 1.396 | 0.875–2.226 | 0.162 |
| MT vs. Med | 0.455 | 0.378 | 1.576 | 0.751–3.310 | 0.229 |
| **TOAST Subtype** |  |  |  |  | 0.567 |
| LAA vs. SUE | 0.66 | 0.749 | 1.935 | 0.446–8.397 | 0.378 |
| CE vs. SUE | 1.024 | 0.827 | 2.784 | 0.550–14.078 | 0.216 |
| SAO vs. SUE | 0.715 | 0.78 | 2.044 | 0.444–9.429 | 0.359 |
| OC vs. SUE | 0.044 | 0.876 | 1.045 | 0.188–5.811 | 0.96 |
| **Medical History** |  |  |  |  |  |
| Hypertension | -0.095 | 0.246 | 0.909 | 0.561–1.472 | 0.699 |
| Diabetes mellitus | -0.527 | 0.274 | 0.590 | 0.345–1.011 | 0.055 |
| Current/ever smoking | -0.229 | 0.254 | 0.795 | 0.483–1.308 | 0.367 |
| Atrial fibrillation | 0.101 | 0.403 | 1.106 | 0.502–2.437 | 0.803 |

**Abbreviations:** NW, normal weight; OW, overweight or obesity; TG, triglycerides; Cho, total cholesterol; HDL-C, high-density lipoprotein cholesterol; LDL-C, low-density lipoprotein cholesterol; NIHSS, National Institutes of Health Stroke Scale; IVT, intravenous thrombolysis; MT, mechanical thrombectomy; Med, standard medical treatment; TOAST, Trial of Org 10172 in Acute Stroke Treatment; LAA, large-artery atherosclerosis; CE, cardioembolism; SAO, small-artery occlusion; OC, other determined etiology; SUE, stroke of undetermined etiology; OR, odds ratio; CI, confidence interval.

**Notes:** Results are derived from an extended multivariable logistic regression model for 90-day excellent outcome (mRS 0–1), including 571 patients. Continuous variables are modeled as per 1-unit increase: age (1 year), NIHSS (1 point), random glucose (1 mmol/L), albumin (1 g/L), and all lipid parameters (1 mmol/L). For categorical variables, the reference groups are as follows: normal weight (for BMI categories), standard medical therapy (for treatment strategy), and SUE (for TOAST subtypes). Binary history variables (hypertension, diabetes mellitus, current/ever smoking, and atrial fibrillation) are compared as yes vs. no. Overweight or obesity (OW) is defined as BMI≥24.0 kg/m^2^ according to Chinese criteria. *p < 0.05.
