# Supplementary material for: Beyond BMI: independent and opposing effects of overweight and obesity and triglycerides on 90-day functional outcomes after acute ischemic stroke
Source: Front Neurol. 2026 Apr 13;17:1782157. doi: 10.3389/fneur.2026.1782157 (PMC13111007; doi:10.3389/fneur.2026.1782157)
Supplement: Supplementary file 2 [file Table_2.docx]

**Supplementary Table S2. Extended multivariable logistic regression model for good outcome (mRS 0–2) at 90 days.**

| **Variable** | **B** | **SE** | **OR** | **95% CI** | ***P* value** |
| --- | --- | --- | --- | --- | --- |
| **Exposure Variables** |  |  |  |  |  |
| TG | 0.328 | 0.178 | 1.388 | 0.980-1.967 | 0.065 |
| OW vs NW | -0.327 | 0.244 | 0.721 | 0.447-1.162 | 0.179 |
| **Other Lipid Profiles** |  |  |  |  |  |
| Cho | -0.004 | 0.197 | 0.996 | 0.676-1.466 | 0.983 |
| HDL-C | -0.015 | 0.036 | 0.985 | 0.918-1.058 | 0.687 |
| LDL-C | -0.151 | 0.193 | 0.860 | 0.589-1.256 | 0.436 |
| **Demographics and Severity** |  |  |  |  |  |
| Age | -0.029 | 0.011 | 0.972 | 0.950-0.994 | 0.012* |
| Male | 0.374 | 0.268 | 1.454 | 0.860-2.459 | 0.163 |
| NIHSS | -0.252 | 0.028 | 0.777 | 0.735-0.822 | 0.000* |
| **Metabolic and Nutritional Markers** |  |  |  |  |  |
| Random glucose | 0.048 | 0.043 | 1.049 | 0.964-1.141 | 0.268 |
| Albumin | 0.006 | 0.021 | 1.006 | 0.965-1.048 | 0.788 |
| **Treatment Strategy** |  |  |  |  | 0.022* |
| IVT vs. Med | 0.612 | 0.271 | 1.845 | 1.085-3.136 | 0.024* |
| MT vs. Med | 0.883 | 0.387 | 2.419 | 1.132-5.167 | 0.023* |
| **TOAST Subtype** |  |  |  |  | 0.599 |
| LAA vs. SUE | -0.578 | 1.009 | 0.561 | 0.078-4.054 | 0.567 |
| CE vs. SUE | -0.650 | 1.061 | 0.522 | 0.065-4.178 | 0.540 |
| SAO vs. SUE | -0.527 | 1.056 | 0.591 | 0.075-4.676 | 0.618 |
| OC vs. SUE | -1.440 | 1.107 | 0.237 | 0.027-2.075 | 0.193 |
| **Medical History** |  |  |  |  |  |
| Hypertension | -0.009 | 0.273 | 0.991 | 0.581-1.691 | 0.974 |
| Diabetes mellitus | -0.963 | 0.310 | 0.382 | 0.208-0.701 | 0.002* |
| Current/ever smoking | -0.647 | 0.283 | 0.524 | 0.301-0.912 | 0.022* |
| Atrial fibrillation | -0.154 | 0.411 | 0.857 | 0.383-1.919 | 0.708 |

**Abbreviations:** NW, normal weight; OW, overweight or obesity; TG, triglycerides; Cho, total cholesterol; HDL-C, high-density lipoprotein cholesterol; LDL-C, low-density lipoprotein cholesterol; NIHSS, National Institutes of Health Stroke Scale; IVT, intravenous thrombolysis; MT, mechanical thrombectomy; Med, standard medical treatment; TOAST, Trial of Org 10172 in Acute Stroke Treatment; LAA, large-artery atherosclerosis; CE, cardioembolism; SAO, small-artery occlusion; OC, other determined etiology; SUE, stroke of undetermined etiology; OR, odds ratio; CI, confidence interval.

**Notes:** Results are derived from an extended multivariable logistic regression model for 90-day good outcome (mRS 0–2), including 571 patients. Continuous variables are modeled as per 1-unit increase: age (1 year), NIHSS (1 point), random glucose (1 mmol/L), albumin (1 g/L), and all lipid parameters (1 mmol/L). For categorical variables, the reference groups are as follows: normal weight (for BMI categories), standard medical therapy (for treatment strategy), and SUE (for TOAST subtypes). Binary history variables (hypertension, diabetes mellitus, current/ever smoking, and atrial fibrillation) are compared as yes vs. no. Overweight or obesity (OW) is defined as BMI≥24.0 kg/m^2^ according to Chinese criteria. **P* < 0.05.
