# Supplementary material for: Beyond BMI: independent and opposing effects of overweight and obesity and triglycerides on 90-day functional outcomes after acute ischemic stroke
Source: Front Neurol. 2026 Apr 13;17:1782157. doi: 10.3389/fneur.2026.1782157 (PMC13111007; doi:10.3389/fneur.2026.1782157)
Supplement: Supplementary file 3 [file Table_3.docx]

**Supplementary Table S3. Unweighted and inverse probability–weighted (IPW) multivariable logistic regression models for excellent outcome (mRS 0–1) at 90 days.**

| **Variable** | **Adjusted OR** | **95% CI** | **p value** | **Adjusted OR (IPW model)** | **95% CI (IPW)** | ***P* value (IPW)** |
| --- | --- | --- | --- | --- | --- | --- |
| OW | 0.604 | 0.396–0.922 | 0.020 | 0.611 | 0.402–0.929 | 0.021 |
| TG | 1.401 | 1.090–1.801 | 0.009 | 1.384 | 1.082–1.770 | 0.010 |
| Age | 0.976 | 0.958–0.995 | 0.012 | 0.977 | 0.959–0.995 | 0.013 |
| NIHSS | 0.773 | 0.735–0.814 | <0.001 | 0.776 | 0.738–0.816 | <0.001 |

**Abbreviations:** OW, overweight or obesity; NW, normal weight; TG, triglycerides; NIHSS, National Institutes of Health Stroke Scale; IPW, inverse probability weighting; OR, odds ratio; CI, confidence interval; IVT, intravenous thrombolysis; MT, endovascular thrombectomy.

**Notes:** Adjusted odds ratios and 95% confidence intervals are derived from multivariable logistic regression models for excellent outcome (mRS 0–1) at 90 days. The left part of the table presents an unweighted model including age, sex, baseline NIHSS score, serum triglycerides, weight status (OW vs. NW), and acute treatment modality (IVT, MT, or standard medical therapy, used as the reference category). The right part shows the same model re-estimated using stabilized IPW to assess robustness to potential selection bias from loss to follow-up or missingness. For clarity, only the four covariates of primary interest (OW vs. NW, TG, age, and NIHSS) are displayed in the table. Age and NIHSS are modeled per 1-unit increase; TG is modeled per 1 mmol/L increase; OW vs. NW is defined according to Chinese BMI criteria.
